# MRI-derived PRECISE scores for predicting pathologically-confirmed radiological progression in prostate cancer patients on active surveillance

**DOI:** 10.1007/s00330-020-07336-0

**Published:** 2020-11-16

**Authors:** Iztok Caglic, Nikita Sushentsev, Vincent J. Gnanapragasam, Evis Sala, Nadeem Shaida, Brendan C. Koo, Vasily Kozlov, Anne Y. Warren, Christof Kastner, Tristan Barrett

**Affiliations:** 1 CamPARI Prostate Cancer Group, Addenbrooke’s Hospital and University of Cambridge, Cambridge, UK; 2 Department of Radiology, Addenbrooke’s Hospital and University of Cambridge, Cambridge, UK; 3 Department of Urology, Addenbrooke’s Hospital, Cambridge, UK; 4 Academic Urology Group, Department of Surgery, University of Cambridge, Cambridge, UK; 5 Cambridge Urology Translational Research and Clinical Trials Office, University of Cambridge, Biomedical Campus, Cambridge, CB2 0QQ UK; 6 Department of Public Health and Healthcare Organisation, Sechenov First Moscow State Medical University, Moscow, Russia; 7 Department of Pathology, Addenbrooke’s Hospital, Cambridge, UK

**Keywords:** Prostate cancer, Magnetic resonance imaging, Active surveillance

## Abstract

**Objectives:**

To assess the predictive value and correlation to pathological progression of the Prostate Cancer Radiological Estimation of Change in Sequential Evaluation (PRECISE) scoring system in the follow-up of prostate cancer (PCa) patients on active surveillance (AS).

**Methods:**

A total of 295 men enrolled on an AS programme between 2011 and 2018 were included. Baseline multiparametric magnetic resonance imaging (mpMRI) was performed at AS entry to guide biopsy. The follow-up mpMRI studies were prospectively reported by two sub-specialist uroradiologists with 10 years and 13 years of experience. PRECISE scores were dichotomized at the cut-off value of 4, and the sensitivity, specificity, positive predictive value and negative predictive value were calculated. Diagnostic performance was further quantified by using area under the receiver operating curve (AUC) which was based on the results of targeted MRI-US fusion biopsy. Univariate analysis using Cox regression was performed to assess which baseline clinical and mpMRI parameters were related to disease progression on AS.

**Results:**

Progression rate of the cohort was 13.9% (41/295) over a median follow-up of 52 months. With a cut-off value of category ≥ 4, the PRECISE scoring system showed sensitivity, specificity, PPV and NPV for predicting progression on AS of 0.76, 0.89, 0.52 and 0.96, respectively. The AUC was 0.82 (95% CI = 0.74–0.90). Prostate-specific antigen density (PSA-D), Likert lesion score and index lesion size were the only significant baseline predictors of progression (each *p* < 0.05).

**Conclusion:**

The PRECISE scoring system showed good overall performance, and the high NPV may help limit the number of follow-up biopsies required in patients on AS.

**Key Points:**

*• PRECISE scores 1–3 have high NPV which could reduce the need for re-biopsy during active surveillance.*

*• PRECISE scores 4–5 have moderate PPV and should trigger either close monitoring or re-biopsy.*

*• Three baseline predictors (PSA density, lesion size and Likert score) have a significant impact on the progression-free survival (PFS) time.*

**Electronic supplementary material:**

The online version of this article (10.1007/s00330-020-07336-0) contains supplementary material, which is available to authorized users.

## Introduction

Active surveillance (AS) is now the recommended management option for men with localized and low-risk prostate cancer (PCa) [1, 2]. The aim of AS is to reduce overtreatment whilst appropriately identifying when progression occurs in order to trigger deferred treatment during the window of curability [3].

Multiparametric magnetic resonance imaging (mpMRI) has become established as an integral part of patient selection for AS [2–5]; however, follow-up is typically based on a clinical combination of prostate-specific antigen (PSA), digital rectal examination and re-staging biopsies [3]. The invasive nature of protocol-driven biopsies may limit patient uptake of AS [6, 7], with MRI potentially offering a means to avoid or limit the number of interventions. The role of mpMRI during AS follow-up is evolving; nevertheless, agreement is lacking on what constitutes radiological progression and whether a positive mpMRI can be used as a stand-alone tool to prompt treatment [8]. A key reason for this is a lack of robust published data, in particular due to inconsistent reporting of follow-up mpMRI findings for patients on AS, thus precluding any meaningful analysis and comparison of the data between the studies [8, 9].

In 2016, a panel of experts in urology, radiology and oncology developed the Prostate Cancer Radiological Estimation of Change in Sequential Evaluation (PRECISE) recommendations in order to standardize reporting and to facilitate data collection regarding the natural history of mpMRI findings in men on active surveillance. The cornerstone of the recommendations was a proposed 5-point Likert scoring scale to standardize the language used to convey the likelihood of radiologic progression, potentially removing any ambiguity in this message [8]. However, its clinical utility is yet to be validated. The aim of our study was to assess the value of the PRECISE scoring system in follow-up of prostate cancer patients on AS and its correlation to disease progression. In addition, we investigated the association between baseline clinical and mpMRI features and progression on AS.

## Materials and methods

### Active surveillance enrolment

Patients with newly diagnosed low-to-intermediate-risk prostate cancer who were selected for active surveillance management at our institution were prospectively entered into an AS study from 2011. The local ethics committee waived the need for informed consent for retrospective analysis from this database (Cambridge University Hospital Trust, Cambridge, UK; registration number: 3592). Enrolment criteria included men aged 50–80 years with Gleason 3 + 3 = 6 or Gleason 3 + 4 = 7 with 10% or less Gleason pattern 4 overall (equivalent ISUP grades 1–2), involving < 50% of all cores; with < 50% involvement of any single-core and ≤ 2-core Gleason pattern 4; clinical stages T1–T2; PSA ≤ 20 ng/ml; and who were otherwise medically fit for radical treatment options. Exclusion criteria included diagnosis of PCa but not meeting pathologically defined enrolment criteria, or previous treatment for PCa. A baseline mpMRI was performed at AS entry, either prior to biopsy or following a standard 12-core systematic TRUS biopsy. In cases where there was a discordance between an initial biopsy result and subsequent mpMRI findings, a repeat targeted transperineal (TP) biopsy was performed within 3 months; any patients upgraded on the basis of this biopsy and no longer matching local AS criteria were considered not to have enrolled for AS.

### Active surveillance follow-up and progression

Follow-up protocol incorporated 3-month PSA testing, annual mpMRI and yearly clinic appointments. Re-biopsies were performed at protocol-driven time points (12 months and 36 months) or were triggered earlier by a clinical suspicion for progression based on three consecutive rises in PSA level or suspected MRI progression. This was defined as PRECISE score ≥ 4 or MRI-based criteria (increase in the number of lesions, increase in lesion size or stage progression) for the scans which predated PRECISE scoring system, as previously reported [10]. In cases where an MRI lesion was visible, a targeted MRI-US image-fusion TP biopsy was performed with 2–4 cores per target in addition to acquiring 24 background systematic cores (2 per each of 12 anatomic sectors) [11]. Progression on AS was defined as pathological progression at re-biopsy or stage progression on mpMRI (from T2 to T3). Pathological progression was defined as a Grade Group increase between diagnostic and repeat biopsy and no longer meeting pathological AS enrolment criteria. Patients with evidence of progression but choosing to not undergo treatment and thus changing to watchful waiting management were considered to be progressing from the date of repeat biopsy. Patients leaving the programme without pathological evidence of progression were excluded from analysis, for instance patient choice, or clinician choice based on PSA progression alone, or MRI increase in lesion size with no confirmatory biopsy. To ensure adequate follow-up and outcome evaluation, patients were followed up for a minimum of 12 months after their last MRI.

### Multiparametric MRI

Patients underwent prostate MRI on a 3-T Discovery MR750 HDx or a 1.5-T MR450 scanner (GE Healthcare) using a 16–32-channel coil, respectively (Supplemental Tables 1 and 2). Axial fast spin-echo T1-weighted images of the pelvis, along with T2-weighted fast recovery fast spin-echo images of the prostate, were acquired in the axial, sagittal and coronal planes, with an axial slice thickness of 3–3.5 mm and a gap of 0–0.5 mm. Diffusion-weighted (DW) imaging was performed using a spin-echo echo-planar imaging pulse sequence (slice thickness 3–4 mm; gap 0 mm), with *b* values of 150 s/mm^2^, 750 s/mm^2^, 1000 s/mm^2^ and 1400 s/mm^2^ (additional 2000 s/mm^2^ at 3 T) and apparent diffusion coefficient (ADC) maps automatically calculated. Dynamic contrast-enhanced (DCE) imaging was performed at baseline, but not in follow-up studies.

### Image analysis

All baseline MRIs were reported by two sub-specialist uroradiologists (B.C.K. and T.B.), with 10 years and 13 years of experience in reporting prostate MRI, respectively, and subsequently reviewed in a multidisciplinary team setting. mpMRI findings were evaluated using a Likert scale, which was initially based on the Prostate Imaging–Reporting and Data System (PI-RADS) v.1 structured scoring criteria developed by the European Society of Urogenital Radiology (ESUR) and, after 2015, on version 2, together with clinical information [12, 13]. The final score was defined by combining all scores for T2WI, DWI and DCE sequences as is now recommended in PI-RADS (version 2.1) [14]: 1 = cancer highly unlikely, 2 = cancer unlikely, 3 = equivocal for cancer, 4 = cancer likely and 5 = cancer highly likely. Likert ≥ 3 of any size on baseline imaging was considered to be an MRI-positive lesion for the purposes of subsequent analysis. The prostate volume was calculated by MRI-based prolate ellipsoid formula (three diameters measured directly on the MRI images, volume = length × width × height × *π* / 6). Prostate-specific antigen density (PSA-D) was then calculated using the MRI-derived gland volume and baseline PSA. Index lesion size was defined at baseline mpMRI as the maximum diameter (mm) using axial T2WI.

**Fig. 1:**
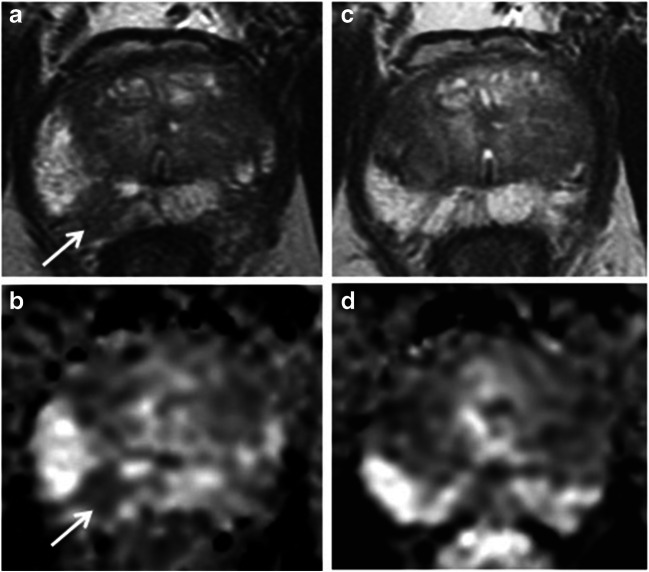
PRECISE score 1. A 62-year-old patient at enrolment, with PSA of 4.53 ng/ml. Top row: T2 axial; bottom row: ADC maps. **a**, **b** MRI shows Likert 3 lesion at the right apex PZ (arrows), with mild-to-moderate restricted diffusion. Targeted biopsy shows Gleason 3 + 3 = 6 in 1/2 cores, 2 mm, 5%. **c**, **d** MRI at 24 months shows almost a complete resolution of T2 intermediate signal intensity change (**c**) and no restricted diffusion on ADC (**d**)

**Fig. 2:**
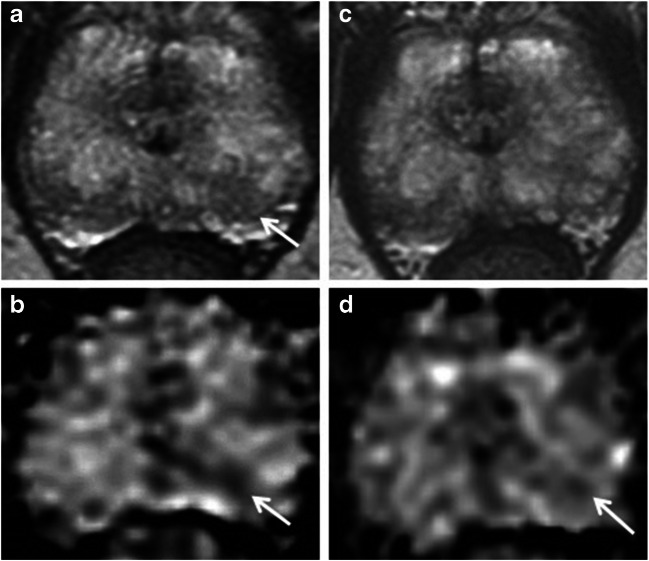
PRECISE score 2. A 70-year-old patient at enrolment, with PSA of 3.4 ng/ml. Top row: T2 axial; bottom row: ADC maps. **a**, **b** 2014 MRI shows Likert 3 lesion at the left apex PZ (arrows). Targeted biopsy shows Gleason 3 + 3 = 6 in 1/3 cores, < 5%. **c**, **d** MRI at 12 months shows more geographical features on T2 (**c**) and reduced conspicuity on ADC (arrow in **d**)

**Fig. 3:**
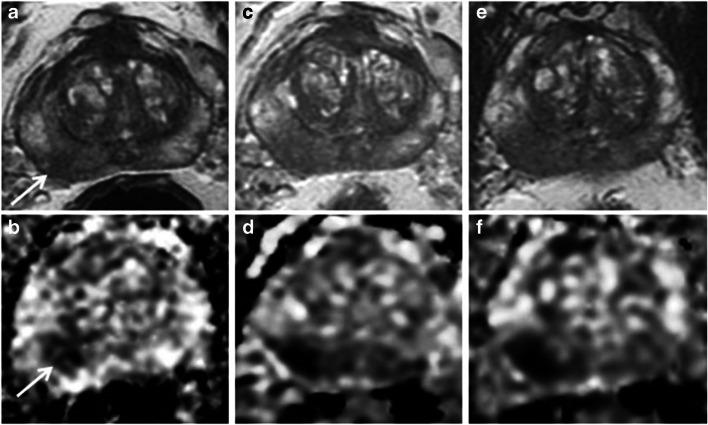
PRECISE score 4. A 66-year-old patient at AS enrolment, with PSA of 3.09 ng/ml. Top row: T2 axial; bottom row: ADC maps. **a**, **b** 2013 MRI shows a Likert 5 lesion in the right mid PZ measuring 12 mm × 7 mm (arrows). Initial biopsy showed Gleason 3 + 3 disease. **c**, **d** 2015 MRI lesion increased to 15 mm × 9 mm (PRECISE 4). **e**, **f** 2017 MRI lesion further increased in size to 17 mm × 10 mm. Targeted biopsy shows Gleason 3 + 4 = 7 up to 6 mm and replacing 85% of both cores. Patient underwent radiotherapy

**Fig. 4:**
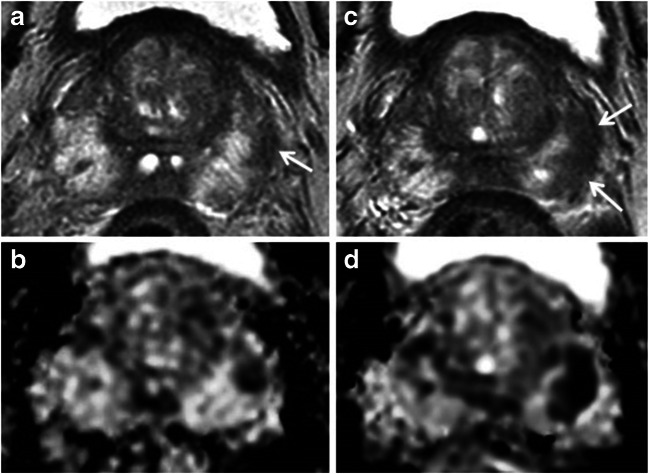
PRECISE score 5. A 68-year-old patient at the time of AS enrolment, with PSA stable at 5.1 ng/ml. Top row: T2 axial; bottom row: ADC maps. **a**, **b** Baseline MRI shows a 9 mm × 7 mm lesion at the left base PZ (arrows). Targeted biopsy showed Gleason 3 + 3 = 6, in 2/2 cores, 45%. **c**, **d** MRI at 30 months shows the lesion has increased in size to 21 mm × 10 mm, with broad capsular contact and irregularity consistent with ECE. Repeat biopsy showed 3 + 4 disease, 62% of cores. Patient underwent hormone and radiotherapy

MRI studies during follow-up were scored on a 5-point scale according to the PRECISE system: (1) resolution of suspicious MRI features (e.g. previous area with restricted diffusion no longer shows it), (2) reduction in volume/conspicuity of MRI features (e.g. reduction in the size of previously seen lesion which remains suspicious for clinically significant cancer), (3) stable MRI appearance (either no suspicious features or all lesions stable in size and appearance), (4) significant increase in the size/conspicuity of features suspicious for PCa (e.g. significant increase in the size of the previously seen lesion or new area of restricted diffusion) and (5) definitive radiologic stage progression (features of extracapsular extension, seminal vesicle involvement or lymph node/bone involvement) [8] (Figs. 1, 2, 3 and 4). PRECISE scores were prospectively reported from June 2016 onwards (*n* = 428). For MRIs performed prior to this period (*n* = 255), PRECISE scores were retrospectively assigned for the 153 cases (22.4% of the cohort total) in which a lesion was present by a single uroradiologist (T.B.).

### Statistical analysis

Statistical analysis was performed using SPSS Statistics 17.0 (IBM Corporation). The Mann–Whitney *U* test was performed to compare continuous baseline parameters (age, PSA, gland volume, PSA density and index lesion size) between patients who showed evidence of disease progression and remained on AS. Pearson’s chi-square test was used for an intergroup comparison of baseline Likert and Gleason scores, treated as ordinal variables. Univariate Cox regression analysis was used to calculate hazard ratios with 95% CIs to identify the prognostic utility of each of the aforementioned baseline parameters. PRECISE scores were dichotomized at a cut-off value of 4 with their diagnostic performance evaluated by the calculation of sensitivity, specificity, positive predictive value (PPV), negative predictive value (NPV) and accuracy per patient level. Kaplan–Meier curves were used to describe progression-free survival outcomes for patients with dichotomized PRECISE scores. Time was measured from the date of enrolment on AS and censored at the date of the last follow-up. *P* values < 0.05 were considered statistically significant.

## Results

**Fig. 5:**
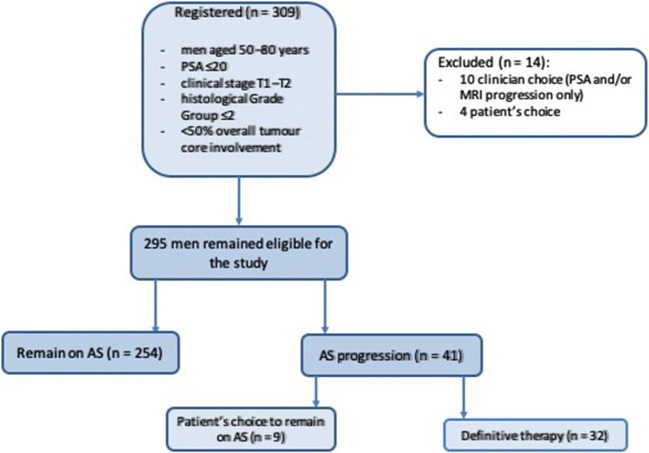
Flowchart for the study inclusion and results

Three hundred nine men were identified from our database, and 14 patients were excluded from analysis due to commencing treatment without evidence of pathological progression: 10 due to clinician choice (PSA and/or MRI progression only) and 4 due to patient choice (Fig. 5). Two hundred ninety-five men were assessed, with the baseline median age of 66 years (IQR 61–69), PSA 5.6 ng/ml (IQR 4–7.9) and median baseline PSA density of 0.10 (IQR 0.1–0.2). Two hundred forty-eight (84%) men had baseline Grade Group 1 (Gleason 3 + 3), and 47 (16%) had Grade Group 2 disease (Gleason 3 + 4) (Table 1). Nine hundred seventy-eight MRI studies were performed, including 683 follow-up studies with PRECISE scores. One hundred thirty-six (46%) cases had a negative MRI at baseline, and of the remaining 159 cases, 48 (16%), 57 (19%) and 54 (18%) patients had an index lesion of Likert score 3, 4 or 5, respectively (Table 2).

**Table 1:** Biopsy Gleason score at baseline and differentiated by those who progressed on active surveillance compared with those without progression

Gleason score	Baseline, *n* (%)^a^	On AS, *n* (%)	Off AS*, *n* (%)	*p* value
3 + 3	248 (84)	216 (85)	0 (0)	
3 + 4	47 (16)	38 (15)	23 (56)	
≥ 4 + 3	0 (0)	0 (0)	11 (27)	0.33

*AS* active surveillance

^a^Baseline Gleason score 3 + 3 versus 3 + 4

*Seven patients (17%) had T3a on MRI/pathology

**Table 2:** Baseline data of the cohort, differentiated by those who progressed on active surveillance compared with those without progression

Parameter	Total (*n* = 295)	On AS (*n* = 254)	Off AS (*n* = 41)	*p* value
Age (years)^a^	66 (61–69)	66 (60–69)	66 (61–69)	0.785
PSA (ng/ml)^a^	5.6 (4–7.9)	5.5 (3.7–7.9)	6 (5.0–7.7)	0.20
PSA-D (ng/ml/cm^3^)^a^	0.10 (0.07–0.16)	0.10 (0.07–0.15)	0.16 (0.1–0.23)	< 0.01
Gland volume (cm^3^)^a^	50.0 (34.0–71.0)	51.5 (36.0–74.3)	41.5 (29.5–55)	< 0.01
Index lesion size (mm)^a^	9 (7–12)	8 (6–11)	11 (8–15)	< 0.01
Follow-up (months)^a^	50 (33–67)	52 (35–68)	39 (25.5–54)	< 0.01
Likert score, *n* (%)				
Likert 1–2	136 (46)	132 (52)	4 (10)	< 0.01^b^
Likert 3	48 (16)	43 (17)	5 (12)	
Likert 4	57 (19)	47 (19)	10 (24)	
Likert 5	54 (19)	32 (12)	22 (54)	

*PSA* prostate-specific antigen, *PSA-D* PSA density

^a^Data presented as median (interquartile range)

^b^Likert 1–2 versus Likert 3–5

### Active surveillance outcome

Of 295 men, 41 (13.9%) progressed at a median time of 39 months (IQR 25.5–54) and 254 (86%) remained on AS at a median follow-up of 52 months (IQR 35–68). Six hundred nineteen biopsies were performed (527 in patients remaining on AS and 92 in patients who progressed). The overall progression-free survival at 5 years was 82.2%. The most common treatment choice for the 41 patients who progressed was brachytherapy (10/41, 24%), followed by hormone and radiotherapy (7, 17%), radical prostatectomy (6, 15%), external beam radiotherapy (3, 7%) and androgen-deprivation therapy (6, 15%); 9 patients (22%) chose not to undergo treatment and thus switched to watchful waiting management.

### Baseline parameters and AS outcome

PSA-D, index lesion size and Likert score were all significantly higher, and gland volume was significantly lower for patients who progressed compared with those remaining on AS (*p* < 0.05, Table 2); baseline Gleason score was not a significant predictor of outcome (*p* = 0.33). Univariate Cox regression analysis revealed that baseline PSA density, index lesion size and baseline Likert score had a significant effect on mean progression-free survival (PFS) time, with hazard ratios of 2.3, 1.1 and 1.9 (*p* < 0.01), respectively (Table 3).

**Table 3:** Univariate Cox regression analysis for evaluating the effect of prognostic factors on progression-free survival time in patients on active surveillance

Parameter	Hazard ratio*	*p* value
Age	1.02 (1.0, 1.1)	0.400
Baseline PSA	1.01 (0.97, 1.06)	0.510
Baseline prostate volume	0.99 (0.98, 1.00)	0.050
Baseline PSA density	2.33 (1.48, 3.67)	< 0.01
First biopsy Gleason score	1.58 (0.73–3.44)	0.250
Index lesion size	1.13 (1.09, 1.17)	< 0.01
Baseline Likert score	1.87 (1.48, 2.36)	< 0.01

*Data in parentheses are 95% CI

### MRI lesion presence

**Fig. 6:**
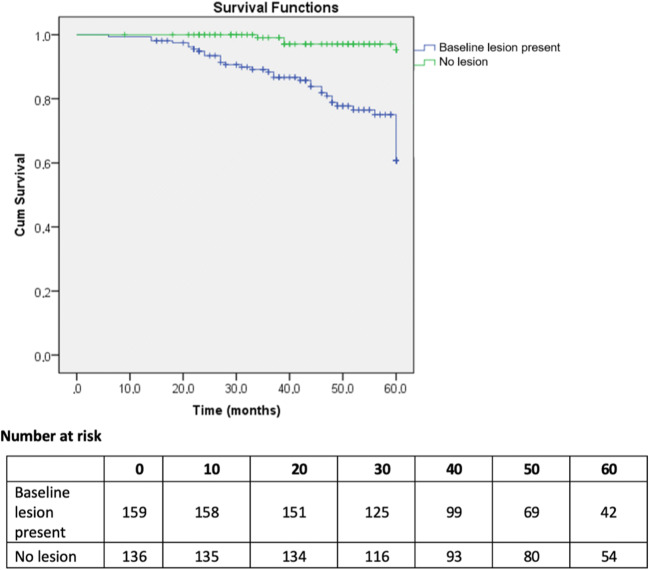
Kaplan-Meier curves for patients with and without MRI-visible baseline lesion

Four of 136 (2.9%) patients with no MRI-visible tumour at baseline progressed, whilst progression was observed in 37/159 (23.3%) patients who had visible disease (Likert ≥ 3) at baseline (*p* < 0.001). The Kaplan–Meier curve showed significantly higher PFS at 60 months for baseline non-visible lesions versus visible lesions at 97.1% and 76.1%, respectively (*p* < 0.01) (Fig. 6). In addition, a significant difference in PFS at 5 years was also observed in patients who only ever scored PRECISE score 3 when dividing these patients into those with no MRI lesion or those having a visible baseline MRI lesion/s at 100.0% versus 91.1% (*p* = 0.001), respectively (Supplemental Figure).

### PRECISE scores and AS outcome

Follow-up PRECISE scores in 683 cases were categorized as PRECISE 1 in 3/683 (0.4%), PRECISE 2 in 39/683 (5.7%), PRECISE 3 in 569/683 (83.7%), PRECISE 4 in 64/683 (9.4%) and PRECISE 5 in 8/683 (1.2%). Of the 41 men who progressed, 2 (4.9%) had PRECISE scores 1–2, 8 (19.5%) had PRECISE score 3, 23 (56.1%) had PRECISE score 4 and 8 (19.5%) had PRECISE score 5 (Table 4). For overall PRECISE scoring, the AUC was 0.82 (95% CI 0.74–0.90), and at a cut-off PRECISE score of ≥ 4, the sensitivity, specificity and accuracy were 75.6%, 88.6% and 86.8%, respectively (Table 5).

**Table 4:** PRECISE scores (*n* = 683) distribution between on-AS and off-AS cohorts

Group	1 (*n*)	2 (*n*)	3 (*n*)	4 (*n*)	5 (*n*)	At least 1 4/5, *n* (%)	Only 3, *n* (%)	At least 1 1/2, *n* (%)
On AS	3	38	511	34	0	29 (11)	192 (76)	33 (13)
Off AS	0	1	58	30	8	31 (76)	8 (20)	2 (2)
Total	3	39	569	64	8	60	200	35

**Table 5:** Performance of the PRECISE scoring system in diagnosing progression during active surveillance

Model	AUC (95% CI)	Sensitivity (%)	Specificity (%)	PPV (%)	NPV (%)	Acc. (%)
PRECISE	0.82 (0.74–0.90)	75.6	88.6	51.7	95.7	86.8
PRECISE for non-visible lesions	0.95 (0.91–1.00)	100	90.1	23.5	100	90.4
PRECISE for visible lesions	0.80 (0.71–0.89)	73.0	86.8	62.8	91.3	83.5

*AUC* area under the curve, *PPV* positive predictive value, *NPV* negative predictive value, *Acc.* accuracy

## Discussion

Our work serves to validate the proposed MRI-based PRECISE scoring system for follow-up assessment of prostate cancer patients on active surveillance. We report a good overall diagnostic performance with a high NPV of PRECISE in predicting progression on AS in a prospective clinical setting. We also demonstrate that diagnostic Likert score, index lesion size and baseline PSA-D are independent baseline predictors of progression on AS. In addition, MRI-visible lesions have a significantly lower progression-free survival than MRI non-visible lesions. Although a marginally higher proportion of Grade Group 2 (17%) versus Grade Group 1 (13%) cancers progressed, this was not statistically significant.

Although previous work has also shown that baseline MRI lesion score and PSA-D are significant predictors of AS progression [15, 16] and evaluation of baseline risk factors is a key in selecting patients for AS and in tailoring follow-up [17], this will not predict the time point when changes may occur, which is potentially offered by MRI-based PRECISE scoring as part of a follow-up programme. PRECISE scoring with a cut-off value of ≥ 4 had an AUC of 0.83 and overall accuracy of 86.8% in predicting AS progression. In addition, the overall NPV for PRECISE scores 1–3 was high at 95.7% with sub-analysis showing that among 41 patients who progressed, only 2 had PRECISE scores 1–2, whilst NPV reached 100% in cases with no MRI-visible lesion. The presence of an MRI lesion is known to predict upgrading in AS patients [18, 19], and it is notable in our cohort that only 2.9% of patients with no lesion progressed compared to 23.3% with an MRI-visible lesion. Conversely, the PPV of PRECISE ≥ 4 was only moderate (51.7%). This is consistent with previous studies showing MRI to have a PPV of 34–69% and an NPV of 70–93% in predicting progression on AS [20–24]; however, a direct comparison is limited by the variable criteria for radiological progression employed by these studies. The high NPV of PRECISE scores 1–3 may reduce the need for follow-up biopsies, whilst the moderate PPV of PRECISE score 4 for predicting true pathological change should, depending on PSA-D and Likert score, trigger either close monitoring or re-biopsy rather than a direct treatment switch.

It is notable that PRECISE scores 1, 2 and 5 were rarely assigned (combined 7.3% of all studies), with score 3 being the most commonly assigned (83.7% of cases). Given the rarity of the extreme scores 1 and 5 (1.6%), the system essentially became a 3-point scoring system, i.e. radiological improvement versus stability versus radiological progression. Of note, the appearance of new lesions is not separately defined within the current PRECISE system and we scored these prospectively as PRECISE-4. Importantly, the PPV of progression for new lesions at 23.5% was noted to be significantly lower than PRECISE scores 4–5 for already existing lesions at 62.8%. Our findings also highlighted that PRECISE 3 has significantly different outcomes for patients with and without a baseline MRI-visible lesion; thus, refinements to the scoring could be considered in the next guideline update.

Another important finding of our study is the low progression rate of 13.9% over a median follow-up of 52 months. This compares favourably with previous studies reporting higher progression rates between 20 and 36% over shorter follow-up periods (1.8–3.9 years) [25–29] and likely reflects the stringent enrolment criteria employed, incorporating MRI and early re-biopsy for discordant historadiological findings. The data from the recent ASIST trial is supportive of our results, and their authors reported a lower rate of pathological progression and 50% fewer AS failures over a 2-year follow-up in the cohort which incorporated baseline MRI and targeted biopsy [30]. Overall, our strategy should limit cases of “pseudo-progression” due to baseline misclassification; therefore, discontinuation of AS likely reflected true pathological progression and enabled more accurate evaluation of the PRECISE system.

Our study benefits from prospective PRECISE scoring in a large AS cohort, with close follow-up and robust outcome data. There are, however, several limitations, including the single-centre and retrospective nature of the analysis. PRECISE scores were prospectively recorded from 2016; however, 22% of studies required a PRECISE score to be retrospectively assigned. This was essential because outcome evaluation requires longer follow-up for AS cohorts. In 8 cases, PRECISE score 5 triggered a direct switch to treatment. One of 8 patients underwent radical prostatectomy where T3a was confirmed at final pathology, whereas 7 of 8 patients were treated by radiotherapy; thus, pathological progression was not definitely confirmed; however, the specificity of MRI for T staging is known to be high [31]. In addition, prospective assignment of PRECISE scores did not allow multi-reader approach for image interpretation and evaluation of inter-reader agreement; however, the main aim of our study was to test the scoring system against real-world outcomes. One of the limitations to this study was the use of different slice thickness and gap parameters at different magnet strengths; however, the protocols remained within the technical specifications of the PI-RADS guidelines and this was done to ensure that optimal imaging quality is achieved on both 3-T and 1.5-T scanning systems. Finally, we employed a Likert scoring system rather than PI-RADS; however, PI-RADS scoring can only be used for baseline evaluation and cannot be used for the follow-up assessment of patients on AS [32], and outcome data in biopsy-naïve patients has shown Likert-based scoring to perform well [33–35]. Future prospective studies assessing the predictive value of PRECISE with standardized AS end-points are required to address these limitations [16].

In conclusion, this study validates the MRI-based PRECISE scoring system in a prospective clinical cohort. Overall performance of PRECISE was considered good in predicting disease progression on AS. Our results show PRECISE scores 1–3 have high NPV which may reduce the need for re-biopsy, whilst PRECISE scores 4–5 have moderate PPV and could trigger either close monitoring or re-biopsy.

## Electronic supplementary material

ESM 1 (DOCX 97 kb)

## Abbreviations

AS: Active surveillance
DWI: Diffusion-weighted imaging
mpMRI: Multiparametric magnetic resonance imaging
PCa: Prostate cancer
PI-RADS: Prostate Imaging–Reporting and Data System
PRECISE: Prostate Cancer Radiological Estimation of Change in Sequential Evaluation
PSA: Prostate-specific antigen
PSA-D: Prostate-specific antigen density
T2WI: T2-weighted imaging
