# Supplementary material for: MRI-derived PRECISE scores for predicting pathologically-confirmed radiological progression in prostate cancer patients on active surveillance
Source: Eur Radiol. 2020 Nov 16;31(5):2696–705. doi: 10.1007/s00330-020-07336-0 (PMC8043947; doi:10.1007/s00330-020-07336-0)

**Supplemental Table 1. 3T MRI protocol**

| **Parameter** | **Axial T2 FSE** | **Axial DWI** | **DCE LAVA** |
| --- | --- | --- | --- |
| TR/TE (ms) | 3743/102 | 3775/85 | 4.3/1.8 |
| FOV, mm | 180 | 280 | 240 |
| Acquisition Matrix | 384x384 | 128x128 | 256x256 |
| Slice thickness, mm | 3 | 3 | 3 |
| Gap, mm | 0 | 0 | 0 |
| b-values | - | 100, 750, 1400, 2000 | - |
| Synthetic b-values | - | 2000, 2500 | - |
| Echo Train Length | 16 | Single shot | 1 |
| Temporal resolution | - | - | 7s |

**Supplemental Table 2. 1.5T MRI protocol**

| **Parameter** | **Axial T2 FSE** | **Axial DWI** | **DCE LAVA** |
| --- | --- | --- | --- |
| TR/TE (ms) | 5048/73 | 3400/62 | 6.2/3.1 |
| FOV, mm | 240 | 280 | 240 |
| Acquisition Matrix | 352x352 | 128x128 | 256x256 |
| Slice thickness, mm | 3.5 | 4 | 4 |
| Gap, mm | 0.5 | 0 | 0 |
| b-values | - | 100, 550, 1000, 1400 | - |
| Synthetic b-values | - | 1400, 2000 | - |
| Echo Train Length | 16 | Single shot | 1 |
| Temporal resolution | - | - | 10s |

**Supplemental Figure. Kaplan-Meier curves for PRECISE 3 score patients with and without MRI visible baseline lesion**


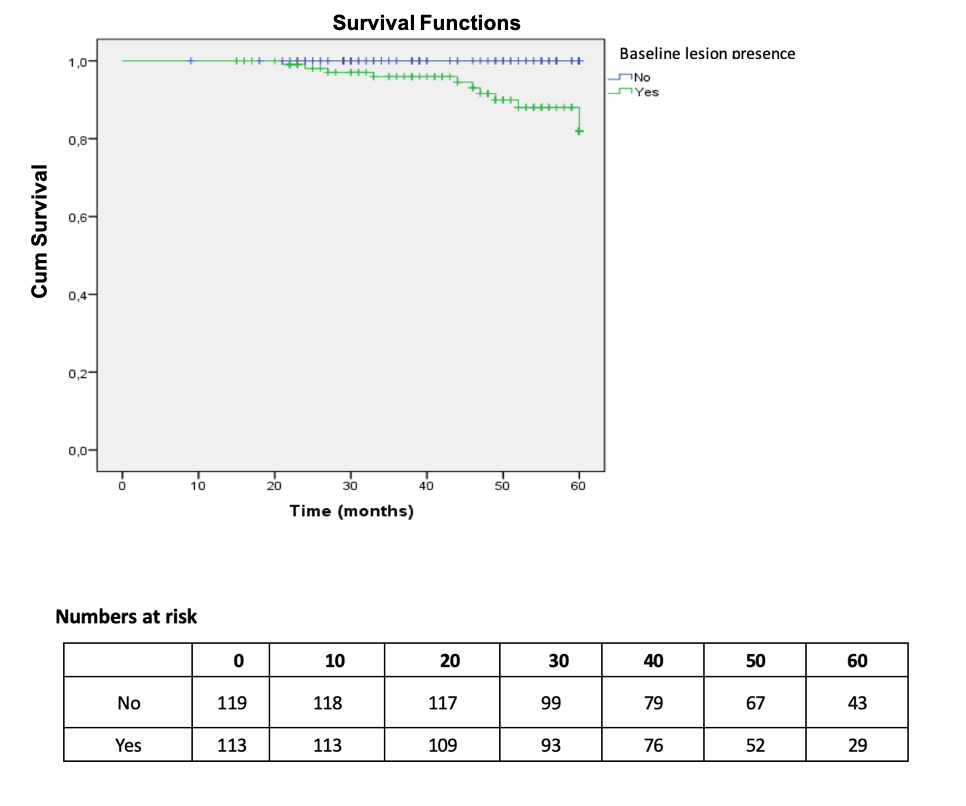

Supplement: Supplementary file 1 — (DOCX 97 kb) [file 330_2020_7336_MOESM1_ESM.docx]
